# Total-body ^11^C-PIB PET/CT imaging of systemic amyloidosis: inter-organ connectivity in cardiac amyloidosis for prognostic insights

**DOI:** 10.1007/s00259-025-07308-w

**Published:** 2025-05-05

**Authors:** Zhihui Hong, Song Xue, Josef Yu, Raffaella Calabretta, David Haberl, Zewen Jiang, Stefan Grünert, Dietrich Beitzke, Andreas Kammerlander, Marcus Hacker, Xiang Li

**Affiliations:** 1 Department of Nuclear Medicine, The Second Affiliated Hospital of Soochow University, Suzhou, China; 2 Division of Nuclear Medicine, Department of Biomedical Imaging and Image-guided Therapy, Vienna General Hospital, Medical University of Vienna, Vienna, 1090 Austria; 3 Division of Cardiovascular and Interventional Radiology, Department of Biomedical Imaging and Image-guided Therapy, Medical University of Vienna, Vienna, Austria; 4 Division of Cardiology, Department of Internal Medicine II, Medical University of Vienna, Vienna, Austria; 5 Department of Nuclear Medicine, Beijing Chest Hospital, Capital Medical University & Beijing Tuberculosis and Tumor Research Institute, Beijing, China

**Keywords:** Systemic amyloidosis, ^11^C-PIB PET/CT, Organ connectivity, Survival analysis

## Abstract

**Background:**

Amyloidosis is a systemic disorder characterized by amyloid fibril deposition in multiple organs, including the heart, brain, liver, kidneys, and lungs, leading to organ dysfunction and affecting survival. In cardiac amyloidosis (CA), where the heart is primarily affected, the broader impact on other organs, particularly the brain, is not fully understood. This study aimed to assess the extent of amyloid deposition across systemic organs in CA, investigate brain-organ, brain-brain and organ-organ connections, and evaluate their effects on patients’ survival.

**Methods:**

A retrospective analysis was conducted on 70 patients, categorized into three groups by biopsy: cardiac amyloidosis (CA) (*n* = 31), non-cardiac amyloidosis (non-CA) (*n* = 12), and no amyloidosis control (NC) (*n* = 27). ^11^C-PIB PET/CT scans were performed, and standard uptake value mean (SUVmean) values were extracted using automated segmentation techniques. Brain-organ, brain-brain, organ-organ connectivity analyses were conducted using Spearman correlation. Survival outcomes and prognostic factors were evaluated using Kaplan–Meier analysis.

**Results:**

Amyloid deposition in CA patients was prominent in the myocardium, liver, and kidneys, reflected by increased SUV_mean_ values. Significant brain-organ connectivity was observed, particularly between the posterior cingulate cortex (PCC), parietal lobes, and myocardial amyloid burden. Additionally, CA patients demonstrated markedly stronger inter-organ connectivity, indicating systemic involvement. In contrast, Non-CA and NC patients demonstrated weaker brain-organ and inter-organ interactions. The CA group exhibited a higher mortality rate, highlighting the severe prognosis, particularly in systemic CA (SCA). Furthermore, elevated amyloid deposition in the bone marrow, lungs and spleen within SCA group was strongly associated with increased mortality.

**Conclusion:**

In CA patients, amyloidosis was associated with extensive systemic involvement, as shown by significant brain-organ and inter-organ connectivity patterns. Findings emphasize that cardiac amyloidosis substantially impacts survival, particularly when accompanied by amyloid deposition in the bone marrow, lungs, and spleen, indicating the need for a comprehensive diagnostic and management approach.

**Clinical trial number:**

Not applicable.

**Supplementary Information:**

The online version contains supplementary material available at 10.1007/s00259-025-07308-w.

## Graphical Abstract

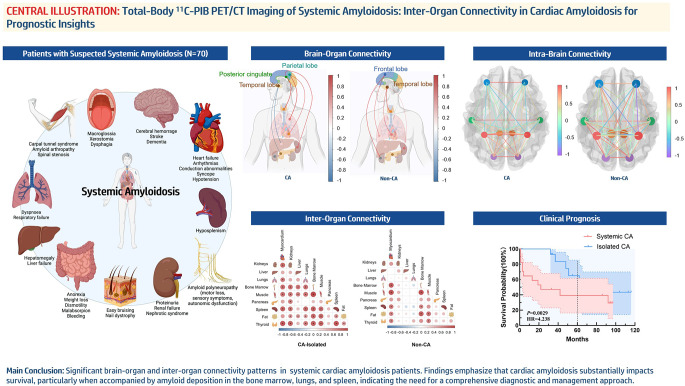

## Introduction

Amyloidosis is a heterogeneous disorder caused by extracellular deposition of misfolded amyloid fibrils in various organs and tissues, leading to progressive organ dysfunction. This complex and variable clinical entity presents significant diagnostic and therapeutic challenges due to its diverse manifestations across multiple organ systems [1–3], including the heart, kidneys, liver, lungs, and brain, leading to substantial morbidity and mortality [4]. Among the subtypes, cardiac amyloidosis (CA) is particularly severe, as amyloid deposition in the myocardium leads to progressive heart failure, arrhythmias, and other cardiac complications, which complicate both symptom management and treatment strategies [5].

Traditionally, amyloidosis research has focused on individual organs to guide therapeutic interventions. However, organ systems are interconnected through complex feedback mechanisms and do not function in isolation. As in the case of CA, amyloid deposition is rarely confined to the heart, and often extends to other organs, indicating a systemic disease process characterized by intricate inter-organ amyloid trafficking or connectivity between the brain and other organs.

Current imaging techniques offer limited insights into these inter-organ and brain-organ connections [6–8]. While a few methods have been applied to study amyloid distribution across individual organs, they fall short of elucidating inter-organ amyloid uptake and the network of brain-organ connectivity.

The 2021 ESC guidelines for cardiac amyloidosis emphasize early systemic evaluation in patients with unexplained cardiac hypertrophy or heart failure with preserved ejection fraction (HFpEF) [9]. These guidelines advocate for multimodal imaging approaches, including amyloid-specific PET tracers, to detect multi-organ amyloid burden and guide risk stratification.

The emergence of ^11^C-Labelled Pittsburgh compound B (^11^C-PIB) positron emission tomography (PET), a radiotracer initially developed for detecting amyloid-β plaques in Alzheimer’s disease [10, 11], offered a promising solution. So far, PIB has been used to detect other types of amyloidosis [12, 13]. PIB PET imaging not only enable visualization but also quantification of amyloid burden in various tissues, providing a comprehensive assessment of the systemic involvement in amyloidosis. By extending its application beyond the brain, whole-body ^11^C-PIB PET imaging can potentially uncover the broader implications of amyloid deposition in peripheral organs and its connectivity with brain function.

This study aimed to explore the systemic nature of amyloidosis, with a a focus on cardiac amyloidosis, using whole-body ^11^C-PIB PET/CT imaging. The primary objective was to investigate the inter-organ connectivity with regard of prognosis, by semi-quantitative analysis the amyloid deposition across various organs. These insights could contribute to a deeper understanding of amyloidosis’s systemic effects and inform more effective diagnostic and management strategies.

## Methods

### Patients characteristics

The clinical characteristics of the participants were summarized in Table 1. We retrospectively included patients with clinical suspicion of amyloidosis based on symptoms, laboratory tests and/or PET imaging suggestive of amyloid infiltration. The inclusion criteria required both: (1) clinical findings consistent with amyloidosis (e.g., heart failure symptoms, thickened ventricular walls on echocardiography, characteristic late gadolinium enhancement (LGE) on MRI, 24 -hour urine protein > 0.5 g/day, total liver span > 15 cm or alkaline phosphatase > 1.5 times the institutional upper limit), and (2) definitive diagnosis required histopathological confirmation through biopsy of at least one affected organ (e.g., endomyocardial, renal, or liver biopsy), with amyloidosis status determined by Congo red staining and immunohistochemical typing. Patients without biopsy confirmation were excluded. Baseline data collected included demographics, clinical history, NYHA score, laboratory tests (e.g., serum-free light chains, proBNP, and Troponin T). The endpoint for the survival analysis was calculated from the date of PIB PET imaging to the date of death or the date of last follow-up (March 1st, 2024). Patients were followed for a minimum of 32 months or until death, with regular clinical assessments, echocardiography, laboratory testing, and additional imaging as needed. Written informed consent was obtained from all participants, and the study was approved by the institutional review board in accordance with the Declaration of Helsinki, and approved by the Vienna General Hospital, Medical University of Vienna Institutional Review Board (Ethic No:1671/2024). All patients provided written informed consent prior to participation.

**Table 1:** Patient characteristics

Characteristic	Overall(*n* = 70)	CA(*n* = 31)	Non-CA(*n* = 12)	NC(*n* = 27)	*p*		
Female	24 (34%)	10 (32%)	4 (33%)	10 (37%)	0.927		
Age (y)	68 ± 12	73 ± 10	62 ± 13	66 ± 12	0.009**		
BMI (kg/m^2^)	27.0 ± 4.8	26.6 ± 3.9	26.7 ± 4.4	27.7 ± 5.8	0.670		
NYHA_score					0.040*		
I	12 (17%)	1 (3%)	6 (50%)	5 (19%)			
II	35 (50%)	18 (58%)	4 (33%)	13 (48%)			
III	23 (33%)	12 (39%)	2 (17%)	9 (33%)			
NT-proBNP (pg/mL)	1894 (489–4230, IQR = 3741)	2311 (1782–4406, IQR = 2624)	255 (53-3440, IQR = 3387)	858 (99-3079, IQR = 2979)	0.001**		
TroponinT (ng/L)	21 (8.5–46.5, IQR = 38)	40.5 (24-68.8, IQR = 44.8)	6 (3–42, IQR = 39)	17.5 (5.8–22.8, IQR = 17)	< 0.001***		
k (mg/L)	21.2 (14-47.3, IQR = 33.3)	18.5 (12.4–29.4, IQR = 17)	20.8 (16.2–62.4, IQR = 46.2)	27.7 (14.4–66.7, IQR = 52.3)	0.178		
λ (mg/L)	36 (20.7–76.5, IQR = 55.8)	41.5 (22.6-113.3, IQR = 90.7)	18.8 (13.6–51.9, IQR = 38.3)	33.9 (24.2–68.8, IQR = 44.6)	0.227		
**PET (SUVmean)**							
Myocardium Liver Kidneys	1.74 ± 1.38 9.74 ± 3.47 4.36 ± 2.56	2.33 ± 1.85 9.17 ± 3.24 5.06 ± 3.23	1.19 ± 0.45 11.47 ± 2.95 3.51 ± 1.19	1.31 ± 0.48 9.63 ± 3.79 3.93 ± 1.90	0.006** 0.062 0.301		
Bone Marrow Lungs Blood Pool Muscles Pancreas Prostate Spleen Fat Thyroid	0.90 ± 0.33 1.58 ± 0.90 1.56 ± 0.51 0.69 ± 0.24 2.59 ± 2.03 2.08 ± 3.01 1.54 ± 0.84 0.39 ± 0.12 1.25 ± 0.75	0.90 ± 0.38 1.78 ± 1.19 1.65 ± 0.57 0.75 ± 0.29 2.85 ± 2.84 3.10 ± 4.33 1.71 ± 0.96 0.37 ± 0.13 1.55 ± 1.02	0.84 ± 0.31 1.44 ± 0.42 1.43 ± 0.39 0.60 ± 0.17 1.99 ± 0.52 1.13 ± 0.38 1.74 ± 1.06 0.31 ± 0.13 1.04 ± 0.33	0.94 ± 0.28 1.41 ± 0.41 1.51 ± 0.48 0.67 ± 0.19 2.55 ± 1.11 1.35 ± 0.71 1.25 ± 0.44 0.35 ± 0.10 1.01 ± 0.22	0.649 0.467 0.501 0.181 0.467 0.202 0.099 0.380 0.018*		
**Cardiac MRI**							
LA (mm)	65 ± 9	66 ± 9	58 ± 9	66 ± 8	0.101		
RA (mm)	64 ± 9	65 ± 8	57 ± 6	65 ± 10	0.116		
LVEF (%)	58 ± 12	57 ± 13	63 ± 8	58 ± 12	0.377		
RVEF (%)	52 ± 11	50 ± 11	55 ± 7	53 ± 12	0.447		
LVEDV (mL)	148 ± 40	146 ± 40	146 ± 28	152 ± 45	0.824		
LVESV (mL)	64 ± 29	66 ± 33	54 ± 17	65 ± 28	0.538		
LVEDD (mm)	46 ± 7	45 ± 6	46 ± 3	48 ± 8	0.333		
RVEDD (mm)	40 ± 6	40 ± 6	37 ± 7	42 ± 6	0.250		
LVSV (mL)	82 ± 25	78 ± 22	87 ± 13	86 ± 32	0.417		
LA area (m^2^)	30 ± 8	32 ± 7	23 ± 8	30 ± 8	0.030*		
RA area (m^2^)	29 ± 8	31 ± 8	23 ± 5	29 ± 9	0.085		
ECV (%)	41 ± 16	51 ± 14	28 ± 7	29 ± 5	< 0.001***		
iLVM (g/m^2^)	91 ± 32	97 ± 32	61 ± 10	87 ± 31	0.167		
**Echocardiography**							
LV (mm)	43 ± 8	40 ± 8	44 ± 6	46 ± 7	0.002**		
RV (mm)	34 ± 7	33 ± 7	29 ± 6	36 ± 6	0.012*		
LA (mm)	60 ± 9	61 ± 8	55 ± 8	60 ± 10	0.168		
RA (mm)	58 ± 9	59 ± 7	52 ± 11	59 ± 11	0.091		
IVS (mm)	17 ± 4	19 ± 4	14 ± 2	15 ± 3	< 0.001***		

CA = Cardiac Amyloidosis; non-CA = non-cardiac amyloidosis; NC = no amyloidosis control; BMI = Body mass index; NYHA = New York Heart Association; NT-proBNP = N-terminal pro-B-type natriuretic peptide; PET = positron emission tomography; SUVmean = standardized uptake value mean; LA = left atrium; RA = right atrium; LVEF = left ventricular ejection fraction; RVEF = right ventricular ejection fraction; LVEDV = Left ventricular end-diastolic volume; LVESV = left ventricular end-systolic volume; LVEDD = left ventricular end-diastolic diameter; RVEDD = right ventricular end-diastolic diameter; LVSV = left ventricular stroke volume; ECV = extracellular volume; iLVM = indexed left ventricular mass; LV = left ventricle; RV = right ventricle; IVS = interventricular septum. Note: * *P*<0.05, ** *P*<0.01, *** *P*<0.001

### ^11^C-PIB PET/CT image acquisition

All patients received an intravenous injection of 555 MBq (15mCi) of ^11^C-PIB. PET imaging was performed using a dedicated PET/CT scanner (Biograph Vision, Siemens, Germany). 1 h after tracer injection, a whole-body low-dose CT scan was first acquired, covering the area from the skull to the upper femur. Following this, PET imaging was conducted with a scan duration of 2 min per bed position. PET images were reconstructed on a 128 × 128 matrix using the ordered subset expectation maximization method (2 iterations, 16 subsets) with a 3.5 mm Gaussian filter.

### Organ auto-segmentation and data extraction of ^11^C-PIB PET/CT image

Fused PET/CT images were visually reviewed on a Hermes workstation (Hermes GOLDLX). For quantitative analyses, Total Segmentator was employed for organ segmentation. Standardized uptake value (SUV) features were then calculated for multiple organs, including the myocardium, liver, spleen, bone marrow, lungs, blood pool, muscles, pancreas, fat, thyroid and various cerebral lobes. For brain amyloid deposition analysis, SUV ratio (SUVr) for each region was calculated by dividing the SUVmean of the target brain region by the SUVmean of the cerebellum.

### Patients classification

Patients included in this study were classified into three main groups based on pathological findings: cardiac amyloidosis (CA), non-cardiac amyloidosis (Non-CA), and a control group without evidence of amyloidosis (NC). Within the CA group, further stratification was performed into two subgroups: isolated cardiac amyloidosis (ICA), defined as amyloidosis exclusively affecting the heart, and systemic cardiac amyloidosis (SCA), characterized by cardiac involvement alongside amyloid deposition in other organs. This classification framework was designed to comprehensively differentiate the disease spectrum and facilitate comparisons between cardiac-specific and systemic amyloid involvement.

### Data analysis

Continuous variables were presented as mean ± standard deviation (SD) for normally or approximately normally distributed data, and median with interquartile range (IQR) for non-normally distributed data. Normality was assessed using the Shapiro-Wilk test. Categoric variables were presented with relative frequencies. Statistical analyses were performed to compare differences across three groups in Table 1. For continuous variables, normality was assessed using Shapiro-Wilk test and homogeneity of variance evaluated with Levene’s test; ANOVA with post-hoc Tukey’s HSD test was used for normally distributed data, while Kruskal-Wallis test followed by Mann-Whitney U tests with Bonferroni correction was applied for non-normally distributed variables. For categorical variables, chi-square test was employed, with Fisher’s exact test (including Monte Carlo simulation for tables larger than 2 × 2) implemented when expected cell counts were below 5. All analyses were conducted using Python (pandas, scipy.stats, stats models) with significance set at *p* < 0.05. These methods directly correspond to the statistical tests implemented in the provided Python code, ensuring methodological consistency between description and analysis. Kaplan–Meier method was conducted to investigate OS outcomes. Statistical significance was established for *p* values of less than 0.05. Statistical analysis of correlation analysis and survival analysis were performed using GraphPad Prism (GraphPad Software).

### Amyloid deposition network analysis

Amyloid network connectivity was defined as spatially coordinated β-amyloid deposition patterns across three biological scales: (a) intracerebral (brain-brain), (b) neuro-systemic (brain-organ), and (c) multisystemic (organ-organ) associations. These connectivity metrics were derived from quantitative ¹¹C-PIB PET imaging using distinct quantification protocols. Cerebral amyloid burden was measured as SUV ratios (SUVr) normalized to cerebellum, while peripheral organ uptake was quantified using SUVmean. Spatial correlation patterns were visualized using hierarchical clustering heatmaps generated via the Hiplot online platform (https://hiplot.com.cn). Inter-organ association strength was assessed through using Spearman correlation coefficients (r_s_), with statistical significance set at *p* < 0.05. Positive correlations indicated concurrent amyloid accumulation across organs, whereas negative correlations suggested potential compensatory mechanism or differences in disease processes. This correlation-based framework represented synchronized pathological protein aggregation across systems, rather than direct anatomical or functional connections.

## Results

### Patients characteristics

Overall, 70 patients were enrolled in this study, stratified into three groups by biopsy: cardiac amyloidosis (CA) group (*n* = 31), Non-cardiac amyloidosis (Non-CA) group (*n* = 12) and non-amyloidosis control (NC) group (*n* = 27). The baseline characteristics of the participants were summarized in Table 1. In the study cohort, there were more male patients (*n* = 46, 66%) than female patients (*n* = 24, 34%). The mean age was 68 ± 12 years. Patients in CA group were significantly older (73 ± 10 years) compared to Non-CA (62 ± 13 years) and NC (66 ± 12 years) groups (*p* = 0.009). Body mass index (BMI) was comparable across the groups, with an overall mean of 27.0 ± 4.8 kg/m^2^. Patients in the CA group showed a higher incidence of heart-related symptoms and related blood test indicators, such as increased NT-proBNP and Troponin T levels, indicating the severity of cardiac involvement in amyloidosis. NT-proBNP levels were significantly higher in the CA group (median [IQR]: 2311 [1782–4406] pg/mL) compared to the Non-CA group (median [IQR]: 255 [53-3440] pg/mL) and NC group (median [IQR]: 858 [99-3079] pg/mL) (*p* = 0.001), reflecting greater cardiac stress.

Echocardiography findings further supported these observations. The CA group exhibited a significantly increased interventricular septum (IVS) thickness (19 ± 4 mm) compared to the Non-CA (14 ± 2 mm) and NC (15 ± 3 mm) groups (*p* < 0.001). Notably, all groups exceeded the normal IVS thickness range (6–10 mm) recommended by the American Society of Echocardiography [14], suggesting potential shared mechanisms of myocardial remodeling beyond amyloidosis (e.g., hypertension or age-related hypertrophy). Additionally, cardiac MRI revealed characteristic late gadolinium enhancement in 90.3% (28/31) of CA patients, compared to 16.7% (2/12) in the Non-CA group and 37.0% (10/27) in the NC group. The high LGE ratio in CA patients aligned with established amyloid infiltration patterns, while the lower rates in Non-CA and NC groups may reflect alternative mechanisms. In terms of PET imaging, the myocardial SUVmean was notably elevated in the CA group (2.33 ± 1.85), indicating amyloid deposition in myocardium.

### Organ involvement by biopsy and ^11^C-PIB PET/CT

On the basis of pathology or autopsy, amyloid deposits were identified in various organs, with the myocardium being the most commonly involved (31 patients, 44.29%), followed by the kidneys (12 patients, 17.14%), bone marrow (12 patients, 17.14%), liver (5 patients, 7.14%), intestine (4 patients, 5.71%), lungs (3 patients, 4.29%), spleen (3 patients, 4.29%), thyroid (2 patients, 2.86%), cervix uteri (1 patient, 1.43%), skin (1 patient, 1.43%), prostate (1 patient, 1.43%), and pancreas (1 patient, 1.43%).

**Fig. 1:**
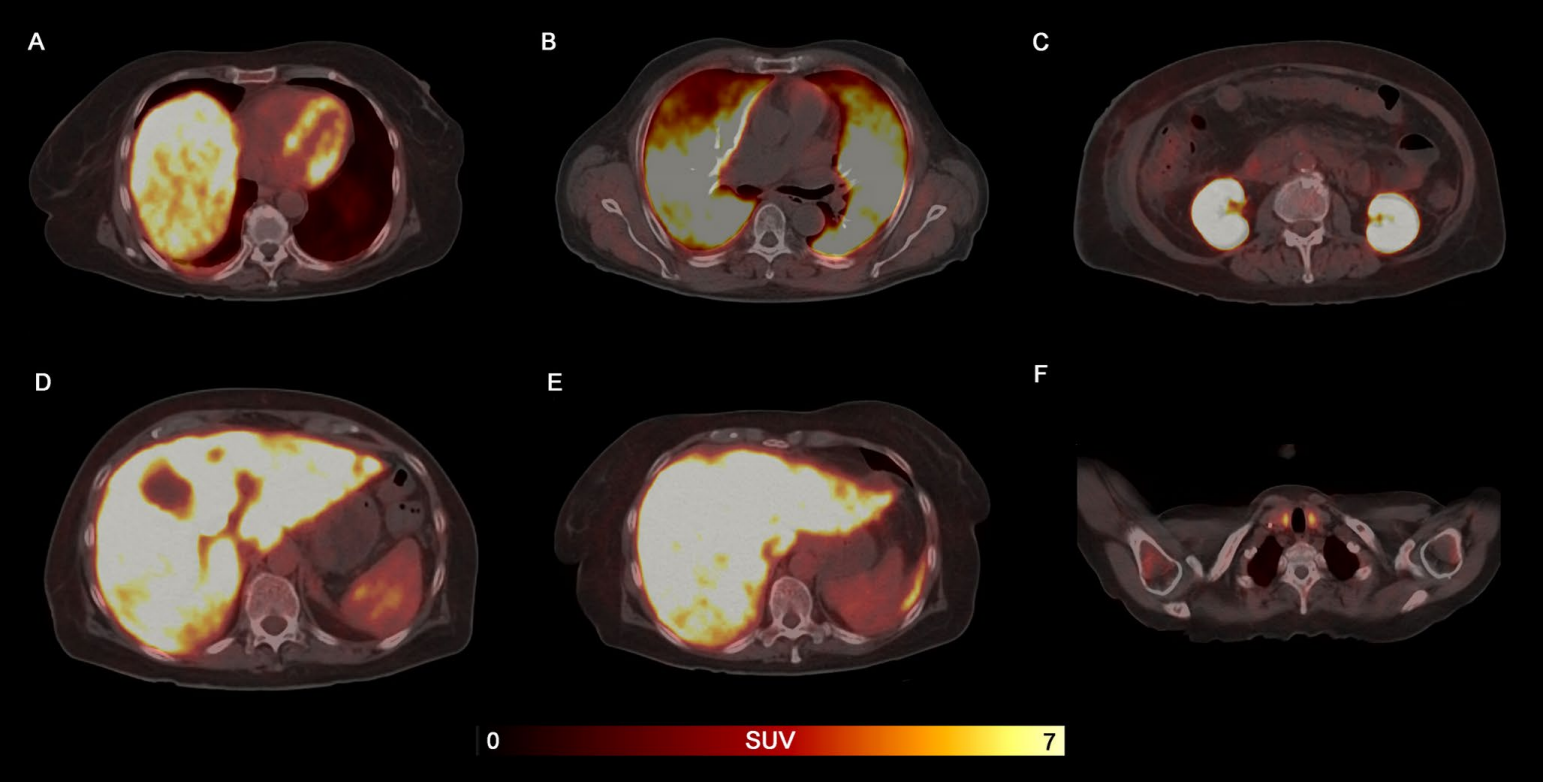
Representative ^11^C-PIB PET/CT images from subjects with biopsy-proven active systemic amyloidosis. Axial fused ^11^C-PIB PET/CT images confirmed diffuse pathological uptake in heart (**A**), lung (**B**), kidney (**C**), spleen (**D**), liver (**E**), and thyroid (**F**)

By visually assessing ^11^C-PIB PET/CT, 11 subjects (15.71%) had a solitary organ involved, 15 subjects (21.43%) had involvement in 2 organs, 8 subjects (11.43%) had 3 organs involved, 12 subjects (17.14%) had 4 organs involved, 4 subject (5.71%) exhibited amyloid deposits in 5 organs, 6 subjects (8.57%) had 6 organs involved. Gastrointestinal tract and bladder involvement were not evaluated in this study due to limitations in visualizing intracavitary radiotracer concentrations. Example images of organ involvement were shown in Fig. 1.

### Non-amyloid biodistribution of ^11^C-PIB PET/CT

In the non-amyloidosis control (NC) group (*n* = 27), physiological ^11^C-PIB distribution exhibited the following patterns (see Supplementary Table S1). Higher homogeneous uptake was observed in the liver (9.63 ± 3.79), a pattern consistent with hepatobiliary excretion. The kidneys demonstrated moderate tracer retention (3.93 ± 1.90), reflecting physiological renal clearance. Pancreas showed relatively higher uptake (2.55 ± 1.11) compared to background organs. Low physiological uptake was observed in most organs, such as brain (0.98 ± 0.08), spleen (1.25 ± 0.44), lungs (1.41 ± 0.41), myocardium (1.31 ± 0.48), bone marrow (0.94 ± 0.28), thyroid (1.01 ± 0.22) and blood pool (1.51 ± 0.48). Negligible retention was found in fat (0.35 ± 0.10), and muscles (0.67 ± 0.19). This physiological profile may provide a baseline for identifying pathological retention in amyloidosis patients.

### Distribution of amyloid de*p*osition in different groups

**Fig. 2:**
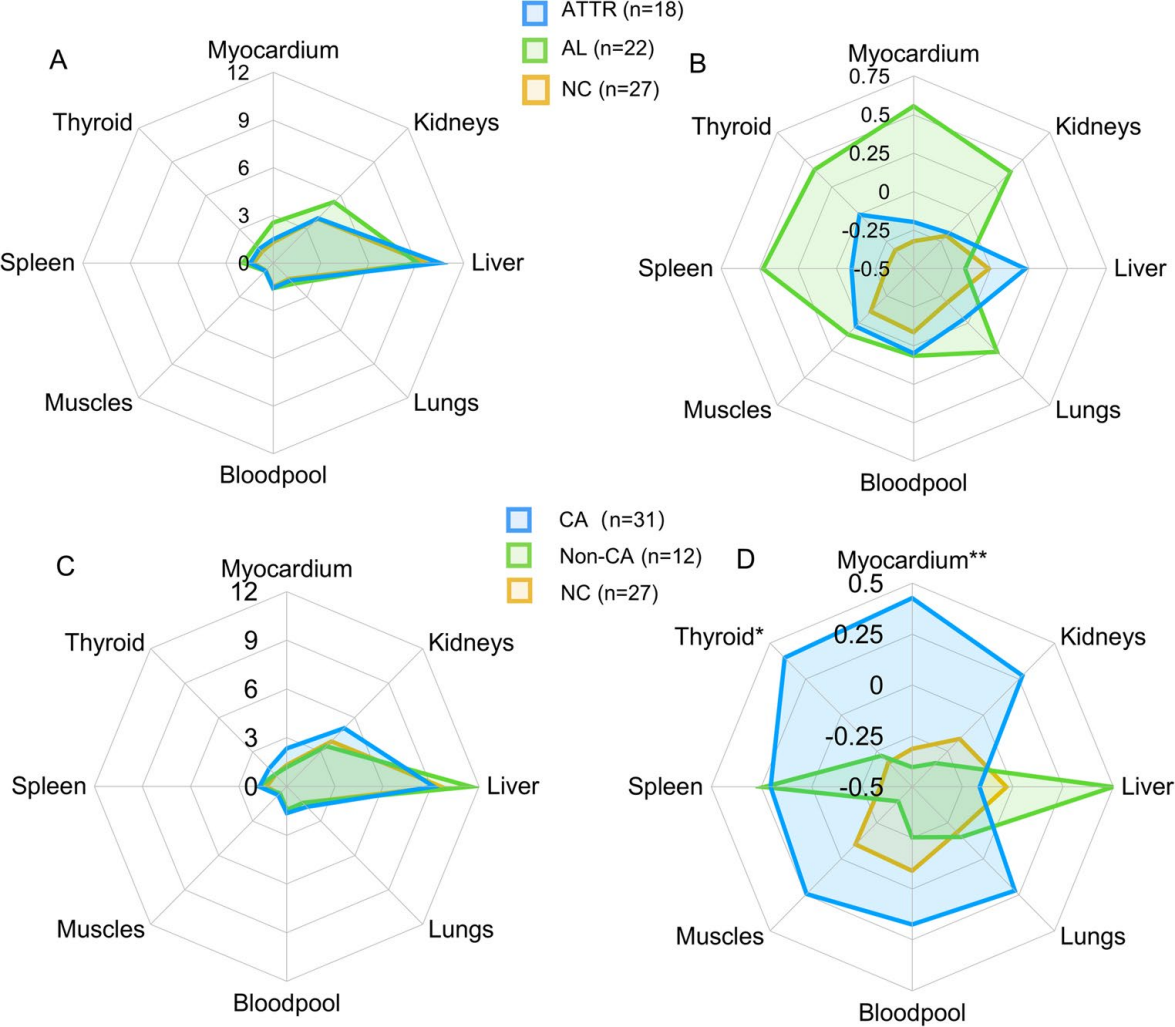
Radar charts display the uptake patterns of ^11^C-PIB across different organ systems for various groups. The axes represent different organ systems, and the data is plotted for ATTR, AL, NC (Panels **A**, **B**), and CA, Non-CA, NC (Panels **C**, **D**), B and D are using Z-normalized. (*p < 0.05, **p < 0.01)

The uptake patterns of ^11^C-PIB across various organ systems were evaluated using radar charts for different groups. The organ-specific analysis of SUV_mean_ values revealed that the distribution among various organs of ^11^C-PIB were different (*p* < 0.001) and predominantly metabolized by the liver and kidneys, consisting with the known metabolic characteristic of this imaging agent (Fig. 2A). To better distinguish the differences of among various amyloidosis subtypes, the SUV_mean_ values were Z-normalized for standardization and visualization. We found that the light-chain (AL) amyloidosis group exhibited notably higher tracer accumulation across multiple organs, with the exception of the liver. While differences were noted in the myocardium, spleen, and thyroid compared to other groups, these differences did not reach statistical significance (*p* > 0.05) (Fig. 2B). Furthermore, when comparing amyloidosis groups (CA, Non-CA, and NC), the myocardium, thyroid and kidneys showed elevated radiotracer uptake in the CA group relative to the other groups (Fig. 2C). Normalization of organ SUV_mean_ confirmed that the CA group demonstrated the highest uptake of ^11^C-PIB, with statistically significant elevations in the myocardium (*p* = 0.006) and thyroid (*p* = 0.018) (Fig. 2D). These results highlighted distinct organ-specific uptake patterns, particularly in the myocardium, that differentiate amyloidosis subtypes and suggest potential for ^11^C-PIB as a diagnostic tool for cardiac involvement.

### Amyloid deposition brain-organ network analysis

Spearman correlation analysis was performed to evaluate amyloid deposition connectivity between brain regions and peripheral organs across the CA, Non-CA, and NC groups, with results visualized using heatmaps.

**Fig. 3:**
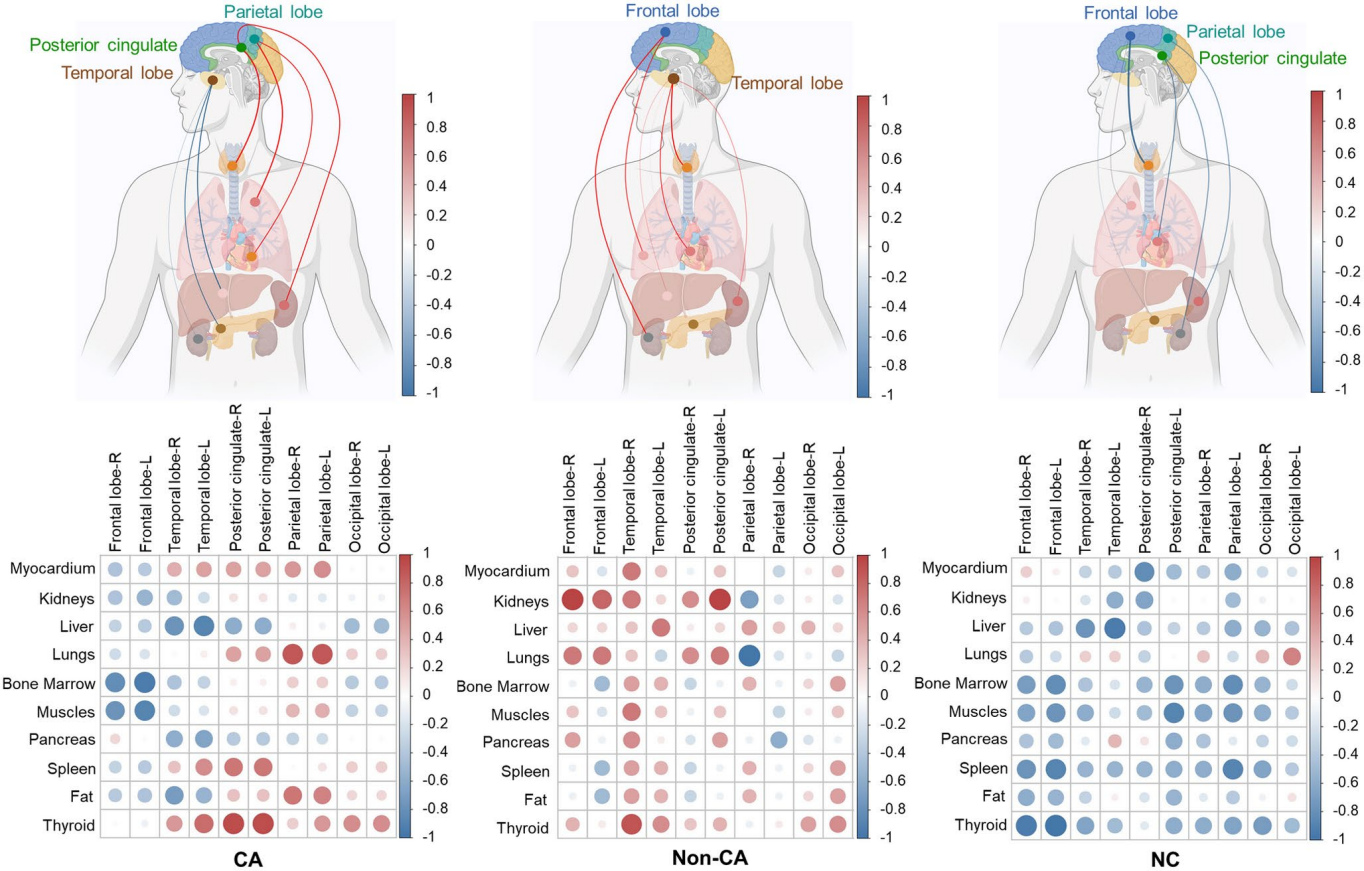
Amyloid deposition connectivity derived from automatically segmented organs and brain lobes where showed the brain-lobe connectivity in patients with CA (*n* = 31), Non-CA (*n* = 12) and NC (*n* = 27)

In the CA group, brain-organ amyloid deposition connectivity revealed a strong positive correlation between several brain regions and peripheral organs, particularly the myocardium, lungs and thyroid. Notable correlations included the parietal lobe with the myocardium (r_s_=0.600, *p* = 0.242) and lungs (r_s_=0.886, *p* = 0.033), as well as posterior cingulate cortex (PCC) and thyroid (r_s_=0.943, *p* = 0.017) (Fig. 3**-CA**). These findings suggested a systemic amyloid burden that links central and peripheral organ systems in cardiac amyloidosis.

In the Non-CA group, while certain organs such as myocardium and thyroid, showed moderate correlations with various brain regions, the overall connectivity patterns were weaker, less consistent, and more variable compared to the CA group (Fig. 3**-Non-CA**). This indicated a reduced and less systemic amyloid deposition in Non-CA patients.

In contrast, the NC group exhibited sparse and predominantly weak or negative correlations between brain regions and peripheral organs, reflected by smaller and less intense color patterns on the heatmap (Fig. 3**-NC**). The minimal connectivity was consistent with the absence of significant amyloid deposition in non-amyloid controls.

These results emphasized that cardiac amyloidosis was not merely a localized pathological condition of the heart but also involved coordinated amyloid deposition across central nervous system and peripheral organs, suggesting a systemic disease process unique to CA.

### Amyloid deposition brain-brain network analysis

**Fig. 4:**
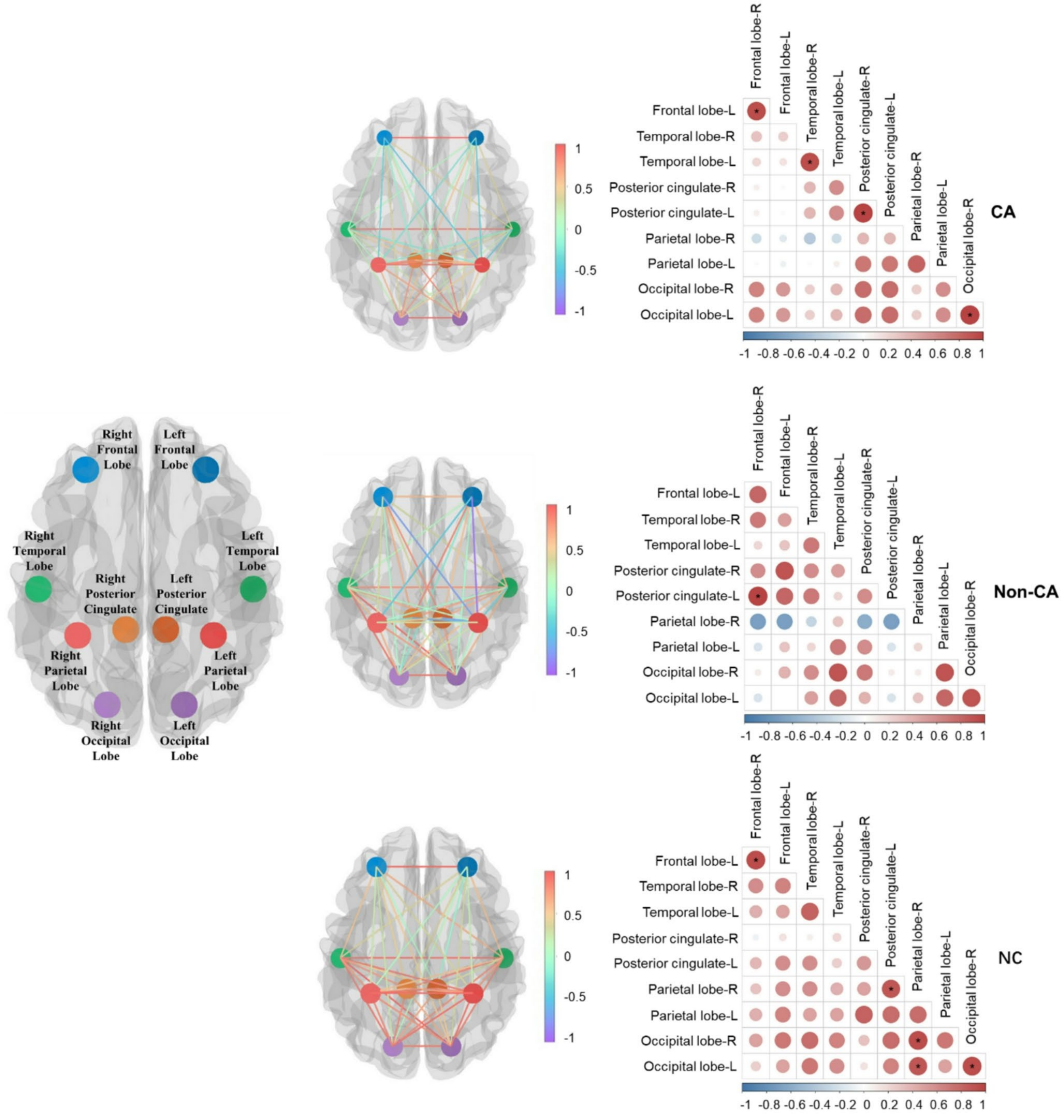
Amyloid deposition connectivity derived from automatically segmented brain lobes where showed the sympathetic-associated brain network connections in patients with CA, Non-CA and NC. Note: the meaning of * symbol indicated statistically significant inter-brain connectivity (Spearman’s *p* < 0.05)

Figure 4 illustrated the amyloid deposition connectivity within the brain, highlighting the sympathetic-associated brain network connections across patients with CA, Non-CA, and NC.

In the CA group, brain-brain network connectivity analysis revealed a strong correlation between the posterior cingulate cortex (PCC) and occipital regions (r_s_=0.771, *p* = 0.103). Additionally, overall connectivity in this group was characterized by weaker interregional correlations compared to NC groups, suggesting disrupted neural network organization (Fig. 4**-CA**). This disruption may reflect neural network reorganization as a compensatory response to systemic amyloid deposition, particularly in cardiac amyloidosis.

In contrast, the Non-CA group exhibited different distinct patterns of brain-brain connectivity. The most prominent connection was observed between the left posterior cingulate cortex (PCC-L) and the right frontal lobe (r_s_=0.900, *p* = 0.017), suggesting a relatively preserved or enhanced network compared to the CA group (Fig. 4**-Non-CA**). This may indicate that amyloid deposition in organs other than the heart did not lead to substantial alterations in brain connectivity patterns.

The NC group displayed a baseline functional connectivity pattern typical of a healthy brain. All the brain regions showed positive connection. Notable correlations were observed between the parietal lobe, occipital lobe, and PCC, reflecting normal physiological neural network interactions and the absence of systemic amyloid burden (Fig. 4**-NC**).

These findings suggested that cardiac amyloidosis, as a more severe systemic form of amyloidosis, may weaken connectivity between brain regions, particularly in networks involving the PCC, occipital, and frontal regions. The resulting disruption in brain connectivity in CA patients highlighted the broader impact of systemic amyloid burden on neural network organization, while the NC group maintain stronger and more consistent connectivity patterns, which provided clues for further understanding the relationship between heart disease and nervous system.

### Amyloid deposition organ-organ network analysis

**Fig. 5:**
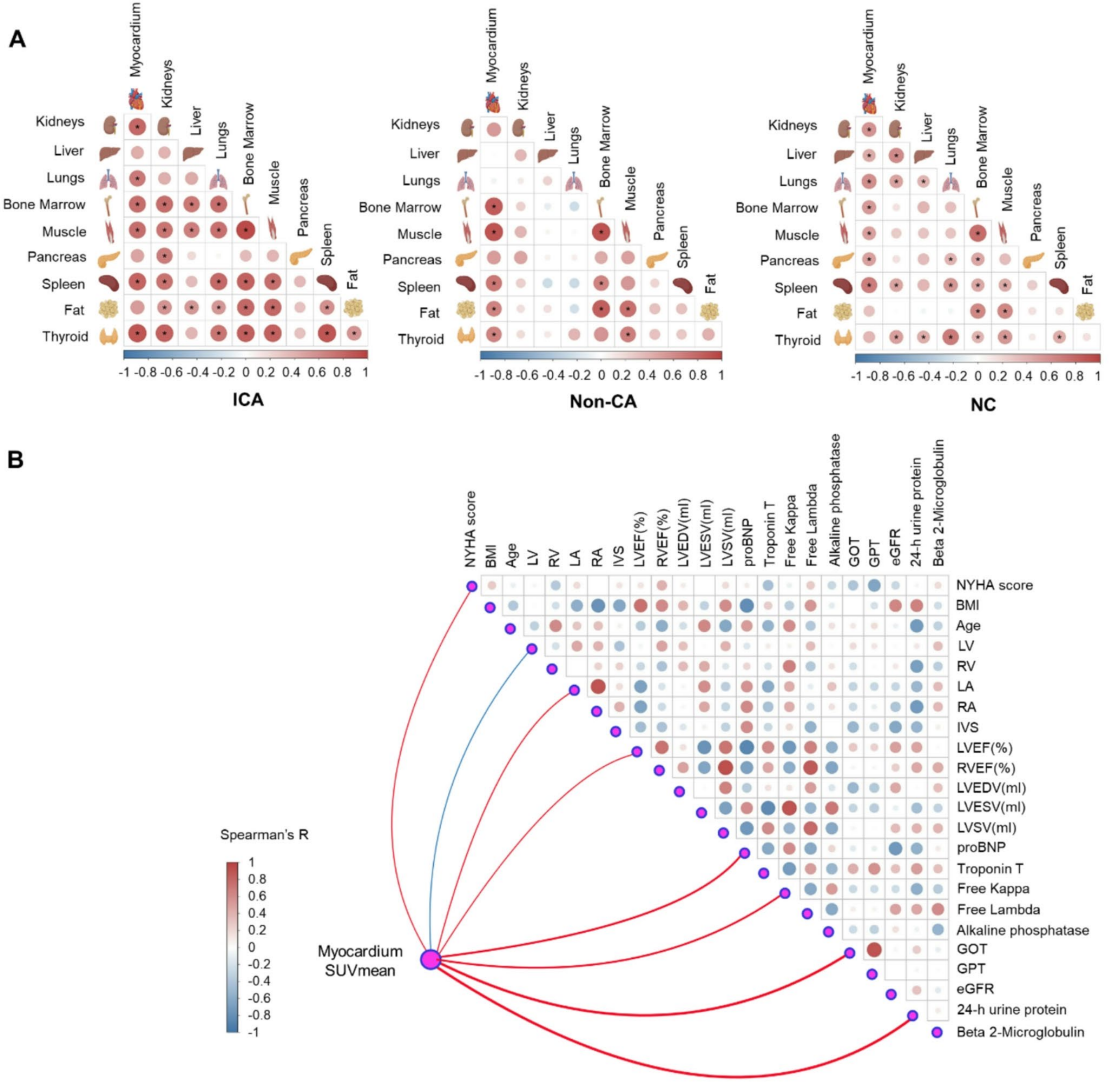
Amyloid deposition connectivity derived from automatically segmented organs where showed the organ network connections in patients with isolated cardiac amyloidosis (ICA), non-cardiac amyloidosis (Non-CA) and no amyloidosis control (NC) (**A**), and the correlation between myocardium SUV_mean_ and clinical parameters within cardiac amyloidosis (CA) group (**B**). Note: the meaning of * symbol indicated statistically significant inter-organ connectivity (Spearman’s *p* < 0.05)

The analysis of organ-to-organ correlations in the ICA group revealed strong positive associations across multiple organs, suggesting a complex network of inter-organ interactions likely influenced by amyloid deposition (Fig. 5A). Significant correlations were observed between bone marrow and muscle (r_s_=0.956, *p* < 0.001), myocardium and thyroid (r_s_=0.886, *p* < 0.001), and spleen and thyroid (r_s_=0.881, *p* < 0.001), indicating enhanced connectivity across organ systems. This pronounced organ-to-organ interaction may reflect early systemic pathological changes characteristic of cardiac amyloidosis. In contrast, the Non-CA group displayed weaker inter-organ correlations, with the strongest link between muscle and bone marrow (r_s_=0.916, *p* < 0.001), while the NC group showed sparse and weak correlations across all organs, with the highest correlation also between muscle and bone marrow (r_s_=0.777, *p* < 0.001).

Additionally, we explored the correlation between myocardial SUV_mean_ and various clinical parameters, including echocardiography, cardiac MR and serological indicators of cardiac, liver and kidney function, within CA group (Fig. 5B). Our analysis revealed positive correlations between myocardial SUV_mean_ and proBNP (r_s_=0.374, *p* = 0.038), GOT (r_s_=0.465, *p* = 0.008), 24-hour urine protein (r_s_=0.48, *p* = 0.024). These findings indicated that myocardium SUV_mean_ may serve as a valuable imaging biomarker for assessing cardiac stress and monitoring disease progression in cardiac amyloidosis patients. Moreover, the observed correlations implied that myocardial SUV_mean_ might be linked to liver and kidney dysfunction, reinforcing the concept of inter-organ connections in CA. This insight highlighted the potential of myocardial SUV_mean_ in assisting early detection and management of related hepatic and renal complications.

These results emphasized that CA patients exhibited more systemic and coordinated amyloid deposition across organs compared to Non-CA and NC groups. Clinical parameters and biomarkers strongly correlate with amyloid burden, indicating their potential as predictors of systemic involvement and disease progression.

### Survival outcomes

**Fig. 6:**
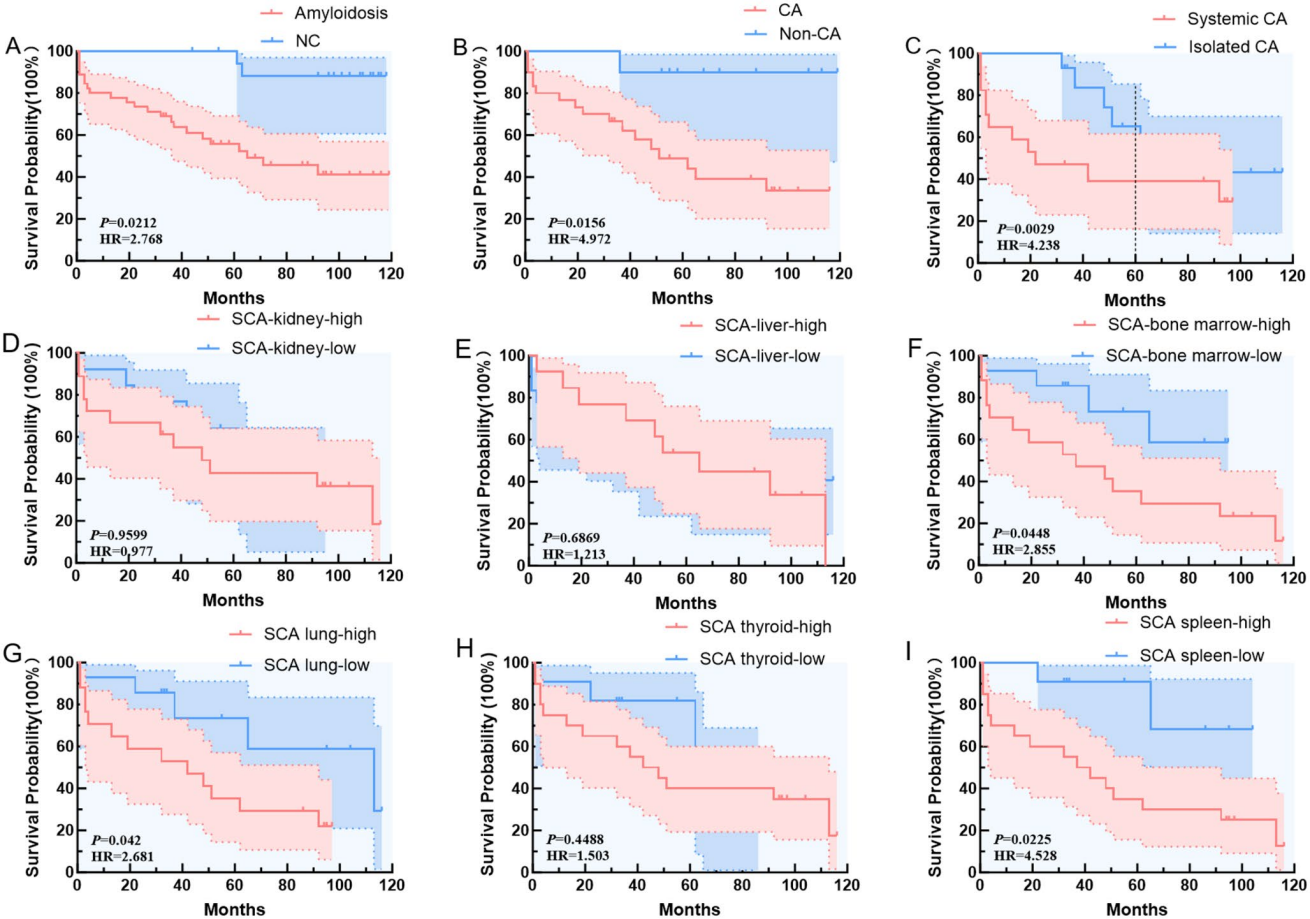
Result of survival analysis between amyloidosis and NC (**A**), CA and Non-CA (**B**), Systemic-CA and Isolated-CA (**C**), SCA-kidney-high (SUVmean > 4.023) and SCA-kidney-low (SUVmean < 4.023) (**D**), SCA-liver-high (SUVmean > 9.394) and SCA-liver-low (SUVmean < 9.394) (**E**), SCA-bone marrow-high (SUVmean > 0.915) and SCA-bone marrow-low (SUVmean < 0.915) (**F**), SCA-lung-high (SUVmean > 1.385) and SCA-lung-low (SUVmean < 1.385) (**G**), SCA-thyroid-high (SUVmean > 1.065) and SCA-thyroid-low (SUVmean < 1.065) (**H**), SCA-spleen-high (SUVmean > 1.369) and SCA-spleen-low (SUVmean < 1.369) (**I**). *p* value less than 0.05 was significant

The survival analysis demonstrated significant differences across various amyloidosis categories. When comparing amyloidosis patients to the NC group, survival rates were significantly lower in the amyloidosis cohort (*p* = 0.0212, HR = 2.768) (Fig. 6A). Further analysis focusing on CA versus Non-CA patients revealed a clear trend of worse survival in the CA group, with statistically significant (*p* = 0.0156, HR = 4.972) (Fig. 6B). Within the CA group, the comparison of systemic cardiac amyloidosis (SCA) and isolated cardiac amyloidosis (ICA) showed significantly reduced survival in the S-CA group, particularly during the first 60 months (*p* = 0.0029, HR = 4.23). After 60 months, the survival curves for both S-CA and I-CA converged, indicating similar long-term outcomes (Fig. 6C). In order to further explore whether the uptake of imaging agents by different organs in the SCA group influenced survival, we divided the cohort into high and low uptake groups based on the median SUV_mean_ of the kidney, liver, bone marrow, lung, thyroid, and spleen, respectively (Fig. 6D-I). Our analysis revealed that high uptake in bone marrow (*p* = 0.0448, HR = 2.855), lung (*p* = 0.042, HR = 2.681) and spleen (*p* = 0.0225, HR = 4.528) had a statistically significant impact on survival. Similarly, we categorized various brain regions by high and low tracer uptake based on the median SUV_mean_ and explored their impact on survival. No statistical difference was found and All *p* values were more than 0.05.

## Discussion

This study provided a comprehensive evaluation of systemic amyloidosis using whole-body ^11^C-PIB PET/CT imaging in 70 patients, divided into subtypes including transthyretin (ATTR) amyloidosis (*n* = 18), light-chain (AL) amyloidosis (*n* = 22), amyloid A (AA) amyloidosis (*n* = 3), and a control group without amyloidosis (NC, *n* = 27). A key finding of this study was the differential pattern of amyloid deposition across various organs and among different amyloidosis subtypes. Amyloid fibrils or their precursors could circulate in the bloodstream and deposit in highly perfused organs. Organs with high vascularization, such as the myocardium, kidneys, liver, spleen, and lungs, may serve as primary targets for amyloid deposition. The study’s finding of bone marrow, spleen, and lung amyloid involvement correlating with poor prognosis indicated that hematopoietic and immune cell interactions might facilitate amyloid propagation.

Distinct organ involvement patterns were observed between ATTR and AL amyloidosis, with ATTR patients exhibiting predominant myocardial deposition, while AL patients demonstrated higher amyloid burden in visceral organs such as the liver and kidneys [15–17]. This was consistent with the known pathophysiology of these subtypes and highlighted the heterogeneity in systemic amyloidosis. The three AA amyloidosis patients, although limited in number, highlighted the diversity of amyloidosis manifestations. It showed a unique pattern of amyloid deposition primarily affecting the kidneys, reflecting the common renal involvement associated with AA amyloidosis [18]. Notably, among the 18 patients with ATTR amyloidosis in our cohort, genetic testing revealed only one case of variant ATTR (ATTRv), characterized by a pathogenic TTR variant, while the remaining 17 cases were classified as wild-type ATTR (ATTRwt).This striking imbalance reflected the epidemiological predominance of ATTRwt in amyloidosis populations, as reported in prior registries [19]. However, the limited sample size of ATTRv precluded robust statistical comparisons between subtypes.

While IVS thickening was a hallmark of cardiac amyloidosis, the observed hypertrophy in Non-CA and NC groups (14–15 mm vs. normal 6–10 mm) may reflect multifactorial mechanisms. Hypertension, present in > 40% of Non-CA/NC patients, was strongly associated with concentric hypertrophy via pressure overload pathways [20]. Concurrently, the cohort’s mean age (> 60 years) may promote age-related myocardial stiffening through fibrosis and collagen deposition [21]. These findings highlighted the need to integrate clinical context (e.g., amyloid biomarkers or multi-organ imaging) to distinguish amyloid-driven remodeling from other etiologies.

The non-amyloid biodistribution of ^11^C-PIB PET/CT demonstrated physiological uptake patterns in the liver and kidneys consistent with prior report in healthy populations [13]. The observed pancreatic uptake, slightly elevated compared to background organs, may reflect intrinsic metabolic activity in acinar cells. Notably, the broad ranges of SUV_mean_ in Supplementary Table S1 were predominantly influenced by two patients with multiple myeloma (MM), whose systemic inflammation or paraprotein deposition likely contributed to diffusely increased tracer retention. Sensitivity analysis excluding MM cases narrowed these ranges substantially (e.g., liver SUV_mean_ reduced to: 4.46–14.65), emphasizing the importance of excluding plasma cell disorders in future studies. Limitations of this study included the small non-amyloid control cohort (*n* = 27), the lack of true healthy volunteers (all controls had clinical indications for imaging), and potential confounding effects of MM-related processes on physiological uptake. Larger, rigorously curated cohort are needed to establish robust normative ^11^C-PIB biodistribution ranges.

The brain-organ connectivity was notably elevated in CA patients, particularly between the parietal lobes and myocardium, lungs, PCC and thyroid. These correlations suggested that systemic amyloidosis may contribute to neurocognitive impairments, potentially due to the impact of cardiac dysfunction on cerebral blood flow and brain metabolism. As well as amyloid deposits in cardiac tissues often extend to the autonomic innervation of the heart, particularly affecting the sympathetic and parasympathetic fibers [22]. In addition, amyloid deposition can interfere with the synthesis, release, and reuptake of neurotransmitters. This can lead to remodeling of neural circuits as the heart and central nervous system attempt to adapt to decreased autonomic input. This finding was consistent with earlier studies that have shown a relationship between cardiovascular disease and cognitive decline in systemic amyloidosis [23]. It suggested that cognitive assessments should be integrated into the routine clinical management of CA patients, as amyloidosis may have greater neurocognitive impacts than previously recognized. The altered brain-organ connectivity patterns in the CA group further suggested that amyloid deposition may lead to compensatory mechanisms or pathological interactions in the brain. These changes could contribute to cognitive impairments commonly seen in CA patients, such as memory loss and confusion.

Inter-brain connectivity analysis in CA group showed weaker connectivity when compared other two groups. The strong connectivity between PCC and the occipital region likely reflected a compensatory response to systemic amyloid deposition. The PCC, a central hub in the brain’s default mode network, reorganized its connections under amyloid burden, potentially to maintain cognitive function [24]. This heightened PCC-occipital connectivity may support visual-spatial and cognitive integration, as the brain attempted to adapt to amyloid-induced neural disruptions. Similar compensatory connectivity patterns had been observed in neurodegenerative conditions, where early network reorganization helped sustain cognitive functions before significant decline [25]. In contrast, the NC group showed a stronger network connectivity, the observed inter-brain connections were mainly concentrated between the parietal lobe, occipital lobe, and PCC, which reflected a uniform distribution of β-amyloid at low physiological levels and consistent functional connectivity within the brain. These findings differed from those of Liping Fu et al., who found minimal brain connectivity in healthy individuals using PIB-PET, possibly due to differences in sample size and age demographics in our cohort [26].

Inter-organ connectivity analysis in the CA group revealed significant correlations across multiple organs, highlighting a unique organ-to-organ network that may reflect early pathological changes. This pattern suggested that CA, while primarily affecting the myocardium, may exert systemic effects across multiple organs even before overt clinical manifestations. The association between cardiac amyloidosis and other organs may provide a new perspective on disease mechanisms. Additionally, the positive correlation of myocardial amyloid with clinical markers such as proBNP, GOT, and 24-hour urine protein reinforced the hypothesis of CA as a systemic disorder with cardiac, hepatic, and renal involvement [27]. These findings emphasized the importance of ^11^C-PIB whole-body imaging in CA, addressing both direct cardiac impacts and broader systemic effects, which may guide early diagnostic and therapeutic interventions.

The survival analysis revealed substantial prognostic differences across various forms of amyloidosis. Patients with amyloidosis had significantly lower survival compared to non-amyloid patients, especially those with cardiac amyloidosis (CA). Systemic cardiac amyloidosis (SCA) was associated with worse outcomes than isolated cardiac amyloidosis (ICA) in the first five years, though survival rates equalized beyond this period. The exploration of imaging agent uptake in organs further showed potential predictors of survival. Interestingly, high uptake in bone marrow, lung and spleen of SCA group showed statistically significant impact on survival, suggesting that bone marrow, lung and spleen involvements may play a critical role in disease progression and patient outcomes. As for bone marrow-high uptake group of SCA, only one case was ATTR type, while the remainder were AL type amyloidosis. As a critical site for hematopoiesis and immune regulation, bone marrow dysfunction from amyloid deposition may exacerbate systemic complications, potentially resulting in poorer survival outcomes [28, 29]. In patients with SCA, high uptake of ^11^C-PIB in the lungs can indicate amyloid deposits that disrupted pulmonary function, affected oxygen exchange and blood flow, thereby increasing the patients’ respiratory burden and leading to a worse overal prognosis [30]. The association of high spleen ^11^C-PIB uptake with worse prognosis in patients with SCA may be closely related to factors such as systemic progression of the disease, splenic dysfunction, increased risk of infection, and systemic inflammatory response, which lead to higher mortality rates and poorer survival prognosis.

This Brain-organ amyloid connectivity (e.g., parietal lobes & posterior cingulate cortex correlating with myocardial amyloid burden) suggests a possible role of the autonomic nervous system in amyloid propagation. Amyloid accumulation in sympathetic ganglia or vagal pathways may disrupt neural control of vascular tone, cardiac function, and systemic metabolism, thereby exacerbating multi-organ dysfunction. Amyloid-related neuronal dysfunction may contribute to altered neurotransmitter regulation, potentially impacting peripheral organ amyloid clearance.

While this study provided valuable insights into the systemic nature of amyloidosis, several limitations must be acknowledged. The sample size of research data was relatively small which may restrict the generalizability of our findings regarding normal biodistribution of ^11^C-PIB, cerebral amyloid deposition and its correlation with cognitive impairment. Moreover, when attempting to stratify CA patients into AL and ATTR subgroups, the small number of patients prevented us from conducting a comprehensive brain-body association analysis. Future studies with larger cohorts would be necessary to confirm and expand on these preliminary observations. Additionally, the retrospective nature of the study introduces potential biases, particularly in patient selection and data availability. While organ segmentation and quantitative analyses were performed using automated software, variations in image quality or patient positioning could influence the accuracy of SUV measurements. Moreover, the absence of biopsy-confirmed amyloid involvement in certain organs, particularly the brain, leaved some uncertainty regarding the specificity of the PET findings. Finally, the follow-up period, though sufficiently long for most patients, may not have captured late-onset complications or long-term outcomes in some cases. Future research should aim to include larger, more diverse cohorts and explore the longitudinal changes in amyloid deposition and organ connectivity over time. Multimodal imaging techniques, combined with molecular and functional biomarkers, could offer a more comprehensive understanding of the systemic effects of amyloidosis and identify new therapeutic targets.

## Conclusion

In conclusion, this study highlighted the systemic involvement of amyloidosis, particularly in cardiac amyloidosis, where amyloid deposition significantly affected multiple organs, including the heart, bone marrow and key organs. Our findings revealed strong brain-organ and inter-organ connectivity, highlighting the complex interaction between affected systems. These results suggested that ^11^C-PIB PET/CT imaging could be a valuable tool for early, whole-body detection of amyloid deposits, providing important prognostic insights, therefore may hopefully formulate personalized treatment strategies and improve patient outcomes.

## Electronic supplementary material

Below is the link to the electronic supplementary material.

Supplementary Material 1

## Data Availability

The data underlying this article will be shared on reasonable request to the corresponding author.
