# Correction to: Total-body ^11^C-PIB PET/CT imaging of systemic amyloidosis: interorgan connectivity in cardiac amyloidosis for prognostic insights

**DOI:** 10.1007/s00259-025-07730-0

**Published:** 2026-01-05

**Authors:** Zhihui Hong, Song Xue, Josef Yu, Raffaella Calabretta, David Haberl, Zewen Jiang, Stefan Grünert, Dietrich Beitzke, Andreas Kammerlander, Marcus Hacker, Xiang Li

**Affiliations:** 1 Department of Nuclear Medicine, The Second Affiliated Hospital of Soochow University, Suzhou, China; 2 Division of Nuclear Medicine, Department of Biomedical Imaging and Image-guided Therapy, Vienna General Hospital, Medical University of Vienna, 1090 Vienna, Austria; 3 Division of Cardiovascular and Interventional Radiology, Department of Biomedical Imaging and Image-guided Therapy, Medical University of Vienna, Vienna, Austria; 4 Division of Cardiology, Department of Internal Medicine II, Medical University of Vienna, Vienna, Austria; 5 Department of Nuclear Medicine, Beijing Chest Hospital, Capital Medical University & Beijing Tuberculosis and Tumor Research Institute, Beijing, China

**Correction to: European Journal of Nuclear Medicine and Molecular Imaging (2025) 52:4985–4999**

10.1007/s00259-025-07308-w

The authors regret that the version of the graphical abstract that appeared in the original published article is incorrect. The original graphical abstract contained an unrelated image that was misleading - or something to this effect.

The incorrect graphical abstract appears below:


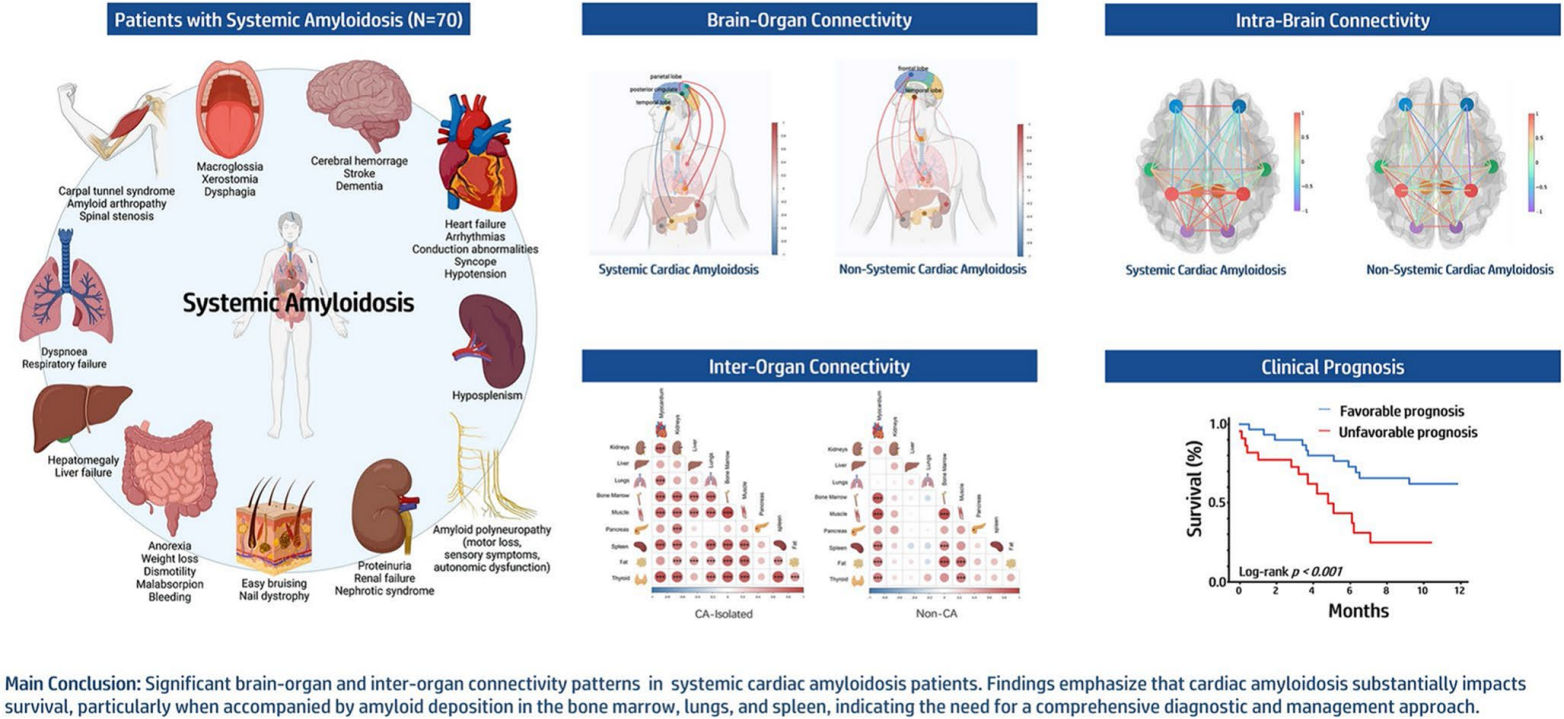


The correct graphical abstract appears below:


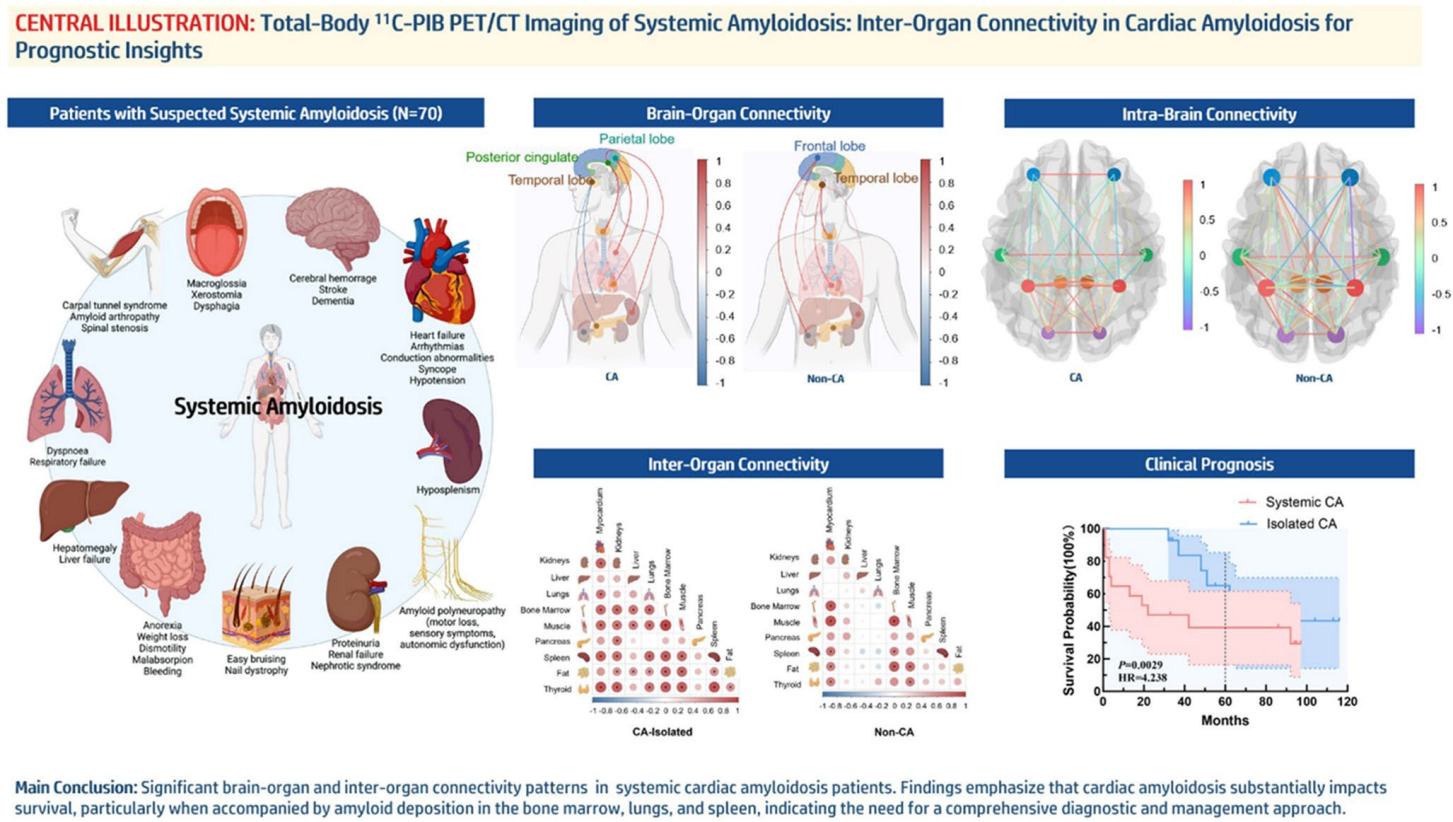


The original article has been corrected.

