# Supplementary material for: Total-body 11C-PIB PET/CT imaging of systemic amyloidosis: inter-organ connectivity in cardiac amyloidosis for prognostic insights
Source: Eur J Nucl Med Mol Imaging. 2025 May 5;52(13):4985–99. doi: 10.1007/s00259-025-07308-w (PMC12589323; doi:10.1007/s00259-025-07308-w)
Supplement: Supplementary file 1 — Supplementary Material 1 [file 259_2025_7308_MOESM1_ESM.docx]

Supplementary Table S1. Physiological ^11^C-PIB uptake in NC group (n=27)

| Organ | SUVmean (mean ± SD) | SUVmean (Range) |  |
| --- | --- | --- | --- |
| Myocardium  Liver  Kidneys | 1.31 ± 0.48  9.63 ± 3.79  3.93 ± 1.90 | 0.53 – 2.61  4.46 – 21.71  0.97 – 8.06 |  |
| Bone Marrow  Lungs  Blood Pool  Muscles  Pancreas  Prostate  Spleen  Fat  Thyroid | 0.94 ± 0.28  1.41 ± 0.41  1.51 ± 0.48  0.67 ± 0.19  2.55 ± 1.11  1.35 ± 0.71  1.25 ± 0.44  0.35 ± 0.10  1.01 ± 0.22 | 0.48 – 1.77  0.59 – 2.91  0.74 – 2.99  0.38 – 1.28  0.92 – 5.20  0.59 – 3.31  0.46 – 2.46  0.16 – 0.56  0.43 –1.34 |  |
| Brain | 0.98 ± 0.08 | 0.90 - 1.10 |  |
